# A randomized, double-blind, placebo-controlled, multicenter study assessing the efficacy of magnesium oxide monohydrate in the treatment of nocturnal leg cramps

**DOI:** 10.1186/s12937-021-00747-9

**Published:** 2021-10-31

**Authors:** Olha Barna, Pavlo Lohoida, Yurii Holovchenko, Andrii Bazylevych, Valentyna Velychko, Iryna Hovbakh, Larysa Bula, Michael Shechter

**Affiliations:** 1 Department of General Practice (Family Medicine), Bogomolets National Medical University, Kyiv, Ukraine; 2 Day Hospital Department, “Artem” Clinic, Kiev, Ukraine; 3 Neurology Department, Clinical Hospital No.3, Kyiv, Ukraine; 4 Family Medicine Department, Railway Clinical Hospital, Odessa, Ukraine; 5 Neurology Department, Clinical Hospital No.9, Kharkiv, Ukraine; 6 “Desna” Clinic, Ternopil, Ukraine; 7 Leviev Heart Center, Chaim Sheba Medical Center, Tel Hashomer, Ramat Gan, Israel; 8 Sackler Faculty of Medicine, Tel Aviv University, Tel Aviv, Israel

**Keywords:** Nocturnal leg cramps, NLC, Magnesium, Food supplement

## Abstract

**Background:**

Magnesium supplements are widely used for prophylaxis and treatment of nocturnal leg cramps (NLC). However, there is little evidence in support of their effectiveness. The main impediment stems from the lack of assessments of cellular absorption. In the current study, we tested the efficacy and safety of a magnesium supplement – magnesium oxide monohydrate (MOMH), for which increased cellular absorption rates were demonstrated in an ex-vivo setting.

**Methods:**

A randomized, double-blind, placebo-controlled multicenter study was conducted in hospitals and outpatient clinics in Ukraine, from February to August 2018. Eligible subjects received a capsule with MOMH 226 mg or placebo, once daily, at bedtime, for a 60-day period. The assessed parameters included frequency and duration of NLC episodes, quality of sleep, NLC-induced pain and quality of life sub-scores. The Fisher’s Exact Test for comparison of groups by categorical variables was used. The Student’s test or Mann-Whitney test were used for between-group comparison at different timepoints. ANCOVA followed by contrast analysis was used for comparison of groups at the end of the study.

**Results:**

175 (81%) out of 216 initially screened subjects completed the study. The number of NLC episodes has significantly decreased by the end of the study period as compared to baseline in both groups (*p* < 0.001 for both). There was a significant between-group difference in the magnitude of reduction in NLC episodes (*p* = 0.01), indicating a higher decrease in the MOMH group as compared to the placebo group (− 3.4 vs − 2.6, respectively). In addition, MOMH treatment resulted in a greater reduction in NLC duration (*p* < 0.007) and greater improvement in sleep quality (p < 0.001) as compared to placebo.

**Conclusions:**

MOMH was shown to be effective in the treatment of NLC as well as safe and well-tolerated.

**Trial registration:**

NCT03807219, retrospectively registered on January 16, 2019.

**Supplementary Information:**

The online version contains supplementary material available at 10.1186/s12937-021-00747-9.

## Introduction

Nocturnal leg cramps (NLC) are a common lower-extremity condition reported by about 50% of adults and ~ 7% of children [1, 2]. Sudden muscle tightening and intense pain result from involuntary and abrupt muscle contractions, typically affecting the calf muscle or the foot [1].

The majority of NLC cases are idiopathic, however, contributing factors have been identified including low levels of certain minerals, such as magnesium, extracellular fluid volume depletion and neurologic, endocrine and metabolic causes [3, 4]. Quinine, the only treatment proven to be effective, has been associated with serious side effects [3, 5–8], leading the FDA to recommend against its usage [3, 9].

Magnesium supplements are commonly used in the treatment of NLC, despite the lack of conclusive evidence for their efficacy [10]. Their effectiveness was demonstrated in a double-blind, randomized, placebo-controlled study of pregnant women [11]. However, other trials [12–14] did not show significant benefits. This could stem from treatment-unrelated effects, such as period-bias, or from a failure to obtain adequate cellular accumulation [15–17].

The potential of magnesium oxide monohydrate (MOMH) to increase intracellular magnesium levels in healthy subjects has been recently demonstrated in a randomized, controlled, crossover study [18]. No significant effects on NLC were found following oral administration of MOMH [19], potentially due to high dropout rate (~ 47%) and a relatively short treatment duration (4 weeks). Indeed, it was reported that to achieve optimal intracellular accumulation, longer administration periods should be implemented [18].

We report the results of a randomized controlled trial testing the hypothesis that MOMH may be effectively and safely used in treating NLC, following 60-day administration.

## Methods

### Standard protocol approvals, registrations, and patient consents

The study protocol, its amendments and the informed consent form were reviewed and approved by Independent Ethics Committees at each site. Reference numbers of approvals and consent forms per site are as follows: 01, 2/1, 158/03, 01/01, 151, 29, and 01/04.

Subjects provided signed informed consent for participation in this study prior to enrollment.

The study was registered in the ClinicalTrials.gov registry. Identifier Number: NCT03807219.

### Trial design

We conducted a prospective, multicenter, randomized, double-blind, placebo-controlled trial to assess the effect of Magnox Comfort – an MOMH supplement, manufactured by Naveh Pharma ltd., on symptoms of NLC. The study was carried out in 7 sites in Ukraine, which included hospitals and outpatient clinics. The study included 2 weeks of screening period, during which potentially eligible subjects were monitored for their NLC episodes, and a 60-day treatment period, during which the enrolled subjects received either active treatment or placebo (1:1). Subjects’ clinical assessments were performed at screening (Visit 0), on Day 1 (Baseline, Visit 1), Day 30 (Visit 2) and Day 60 (End of Treatment, Visit 3). The subjects, the PIs and all the other research personnel, including the statistician, were blinded to the received treatment.

### Setting and participants

A total of 216 subjects who have been diagnosed with NLC, entered the screening period of the trial. 184 subjects were randomized, and 175 subjects completed the study. The NLC frequency of two subjects in the MOMH group numerically exceeded the cumulative NLC duration throughout all visits, namely, the accumulated NLC duration (in seconds) was shorter than the total number of NLC episodes. Their data were therefore, excluded from all the analyses except for safety, resulting in 86 subjects in the MOMH group.

Inclusion criteria were males and females aged ≥45 years with neurologically intact function of both lower extremities, for whom NLC was established with at least 4 NLC episodes during the screening period.

Exclusion criteria included alcoholism or drug addiction, and the following medical conditions: hyper- or hypothyroidism, renal insufficiency (glomerular filtration rate [eGFR] < 60 ml/min/1.73 m^2^), past surgery on the lower extremities, endovascular arterial reconstruction, sympathectomy, deep vein thrombosis, periodic limb movement syndrome, restless legs syndrome, lameness, cramps associated with exercise, hypnagogic jerks, myositis, myalgias, peripheral neuropathy or symptoms of severe lower limb ischemia. Subjects who were prescribed and taking statins, proton pump inhibitors, medications or dietary supplements for the treatment of NLC such as carisoprodol, diltiazem, gabapentin, verapamil, quinine, vitamin B12, vitamin D, vitamin E, vitamin B6 or magnesium, during 30 days prior to screening initiation, were excluded. Planning a pregnancy, pregnancy and breastfeeding were also part of the exclusion criteria.

### Randomization and intervention

Eligible subjects were randomly allocated, in a 1:1 ratio, to the MOMH or the placebo group, using a random numbers generator. MOMH 226 mg or placebo were taken orally once daily at bedtime for a period of 60 days. The appearance, color, smell and taste of the MOMH and placebo capsules were identical to maintain treatment blinding. On each visit, subjects returned the unused product and received a new pack for the next between-visits period. Compliance was assessed by counting the unused capsules. As per the protocol, low compliance was identified if less than 75% of the prescribed dose was taken.

### Outcomes and follow-up

The primary outcome of the study was the mean difference in the effect of MOMH on the frequency of NLC, as compared to placebo, during the study. Secondary outcomes included the differences between the MOMH group and the placebo group in the duration of NLC, the severity of NLC-associated pain, change in the quality of sleep, change in the quality of life and the dropout rate during the study. The primary outcome was assessed by comparing the change in the number of NLC episodes per week from Baseline (Visit 1) to Day 30 (Visit 2) and Day 60 (Visit 3, End of Study) between the MOMH group and the placebo group. Within-group comparisons between Baseline and Days 30 and 60 were also performed. Secondary outcomes were assessed by comparing the tested parameters between the MOMH group and the placebo group at Baseline and on Days 30 and 60. Within-group comparisons between Baseline and Days 30 and 60 were carried out as well. Safety outcomes were the frequency and the severity of adverse events, which were monitored throughout the study. Subjects who dropped out prior to study completion were included in the analyses of safety.

### Outcome measurement tools

#### Number of NLC episodes

Subjects monitored the number of NLC episodes on a daily basis via patient diaries. These reports were extracted and the number of NLC episodes per week was calculated at each study visit.

#### Severity and duration of NLC

Subjects monitored the severity of pain induced by NLC as well as the duration of the NLC episodes on a daily basis via patient diaries. Subjects rated the NLC-associated pain using a 0–10 Visual Analogue Scale (VAS) with ‘0’ indicating no pain and ‘10’ – intolerable pain. These reports were extracted and the mean pain scores and mean NLC durations per week were calculated at each study visit.

#### Sleep quality

Subjects monitored their quality of sleep on a daily basis using 0–5 VAS, with ‘0’ indicating complete absence of sleep disorders and ‘5’ indicating severe sleep disturbances. Weekly cumulative sums of the ratings were calculated for each patient and entered into the case report form.

#### Quality of life

Quality of life was assessed using the 36-Item Short Form Health Survey (SF-36) [20, 21], at Baseline and on Days 30 and 60. This survey yields an 8-scale profile of functional health and well-being, as well as psychometrically based physical and mental health summary measures and a preference-based health utility index. SF-36 has been validated in Russian.

### Sample size

Sample size calculation was based on the primary outcome. The maximum range of the number of episodes was estimated to be between 4 (based on the inclusion criterion) and 60 (an episode each night). The estimated standard deviation was ~ 9 (56/6). Assuming a mean reduction of 3 episodes during the study period, a sample size of 86 participants in each group was calculated to provide the study with at least 80% power with a significance level of 5%.

### Statistical analysis

Data analysis was conducted with SPSS Statistical Package version 23 (SPSS Inc. Released 2015. IBM SPSS Statistics for Windows, version 23.0, Armnok, NY, IBM Corp.). Continuous data were expressed as arithmetic means ±standard deviation. Categorical variables were expressed as frequencies. The Fisher’s Exact Test for comparison of groups by categorical variables was used. The Student’s test for independent data or Mann-Whitney test (dependent of normality check with Shapiro-Wilk test results) was used for between-group comparison at different timepoints. ANCOVA followed by contrast analysis (simple contrasts) was used for comparison of groups at the end of the study. ANOVA with two factors (“time” as fixed effect and “subject” as random effect) followed by contrast analysis (simple contrasts) was used to evaluate the changes over time. Distribution of ANOVA and ANCOVA residuals was checked for normality by Shapiro-Wilk test. If non-normal distribution was identified, ANOVA or ANCOVA on ranks was used [22, 23]. For Shapiro-Wilk test the significance level was set to 0.01, for all other tests it was set to 0.05.

### Data availability statement

Any additional data, collected but not included in this paper, including study protocol and statistical analysis plan, will be de-identified and shared upon request from any qualified investigator.

## Results

**Fig. 1:**
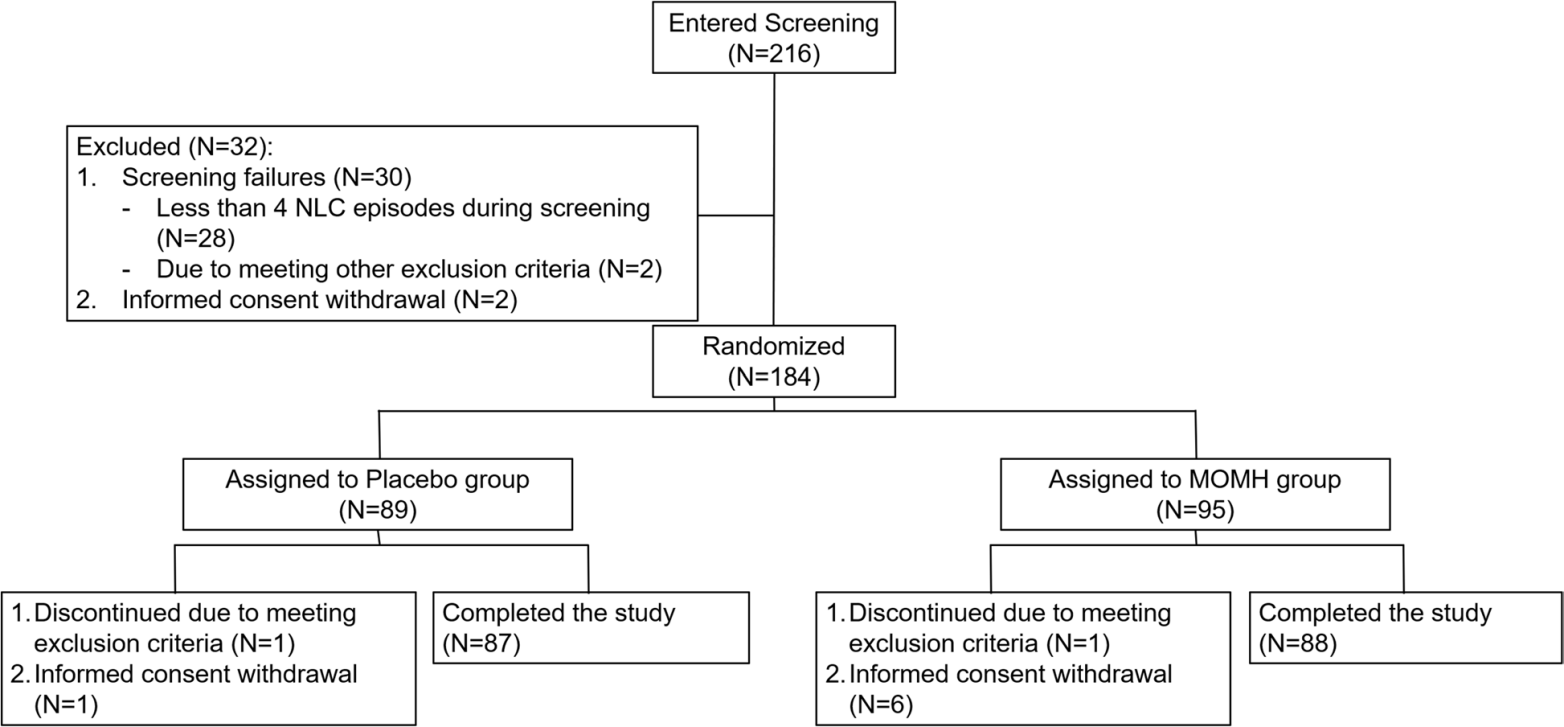
Study Flow Diagram

216 subjects who have been diagnosed with NLC, were recruited from hospital wards and outpatient clinics and entered the screening period of the trial. 28 subjects were subsequently excluded due to having less than 4 NLC episodes during the 2-week screening period. 2 additional subjects were excluded due to meeting other exclusion criteria and 2 subjects withdrew their consent. 184 were randomized into the placebo (*N* = 89) and MOMH (*N* = 95) groups. After treatment initiation, 2 subjects were excluded due to meeting exclusion criteria and 7 subjects withdrew their consent. 175 subjects, 87 in the placebo group and 88 in the MOMH group, completed the study. The study flow diagram, per CONSORT guidelines, is presented in Fig. 1. The study was conducted from February 2018 to September 2018.

Baseline characteristics per group are presented in Table 1. The groups were similar with respect to gender distribution, age, height, weight and body mass index (BMI). Physiological parameters that included systolic and diastolic blood pressure (SBP and DBP, respectively) and heart rate, were similar between the groups as well. Importantly, there were no between-group differences in NLC frequency, duration and pain, sleep quality and the quality of life evaluations at Baseline.

**Table 1:** Baseline characteristics by group

	MOMH group *N* = 86	Placebo group *N* = 87	*P* Value
Demographics			
Gender Female n (%)	65 (75.6)	64 (73.6)	0.862
Age (years) mean (±SD)	57.3 (±10.7)	57.1 (±10.2)	0.955^a^
Weight (kg) mean (±SD)	74.9 (±13.0)	74.9 (±13.9)	0.995
Height (cm) mean (±SD)	167.4 (±9.6)	167.4 (±9.3)	0.991
BMI (kg/m^2^) mean (±SD)	26.7 (±4.0)	26.7 (± 4.5)	0.791^a^
Physiological parameters			
Systolic blood pressure (mmHg) mean (±SD)	128.6 (±11.5)	130.0 (±10.3)	0.519^a^
Diastolic blood pressure (mmHg) mean (±SD)	80.0 (±6.9)	79.0 (±6.2)	0.246^a^
Heart rate (beats/min) mean (±SD)	71.5 (±6.5)	71.6 (±6.2)	0.934^a^
Baseline efficacy parameters			
NLC frequency (num/week) mean (±SD)	5.4 (±5.0)	6.4 (±8.4)	0.523^a^
NLC duration (sec/week) mean (±SD)	244.5 (±238.6)	266.5 (±248.9)	0.562^a^
NLC pain (mean VAS/week) mean (±SD)	6.6 (±1.4)	6.7 (±1.4)	0.584
Sleep quality (mean cumulative score/week) mean (±SD)	13.1 (±3.4)	12.5 (±3.7)	0.327
Quality of life subscales:			
- Physical functioning	63.6 (±25.4)	64.8 (±27.5)	0.904^a^
- Role limitation due to physical health	44.5 (±45.3)	46.6 (±42.7)	0.686^a^
- Role limitation due to emotional problems	40.7 (±45.0)	40.6 (±43.6)	0.993^a^
- Vitality	45.9 (±14.5)	45.9 (±12.3)	0.746^a^
- Mental health	50.1 (±13.6)	49.9 (±11.6)	0.944^a^
- Social functioning	61.9 (±16.8)	60.1 (±16.3)	0.572^a^
- Body Pain	47.9 (±17.3)	49.9 (±18.9)	0.640^a^
- General health	44.9 (±12.1)	45.9 (±9.3)	0.551^a^

*BMI* body mass index, *MOMH* magnesium oxide monohydrate, *NLC* nocturnal leg cramps, *SD* standard deviation, *VAS* visual analogue scale

Note: Variables marked with ^a^ were not normally distributed. ANOVA and ANCOVA on ranks were performed for comparisons of these variables

Concomitant illnesses assessed included hypertension, coronary artery disease, cardiosclerosis, osteochondrosis, heart failure, angina pectoris, chronic tonsillitis, chronic cholecystitis, arthrosis, chronic pancreatitis, chronic prostatitis, diabetes mellitus, stomach ulcer, hypertensive heart, chronic sinusitis in remission stage, chronic gastritis, chronic pyelonephritis, chronic cystitis in remission stage, adenoma of prostate, bronchial asthma, varicose disease of the lower extremities, tension headache, dizziness, insomnia, migraine, myopia, neurocirculatory dystonia of the cardiac type, cataract, anxiety disorder, fibroscopic mastopathy, Parkinson’s disease, chronic bronchitis, chronic gastroduodenitis, chronic glomerulonephritis (remission stage), chronic tonsillopharyngitis (remission stage), cerebral atherosclerosis, post-onset (ischemic stroke in 2005) encephalopathy, kidney cyst, climacteric vegetative disorders, constitutional-exogenous obesity (I degree), breast myoma, pre-diabetes (impaired glucose tolerance), reactive arthritis, urinogenic diathesis, chronic dyshidrotic eczema, chronic dyscirculatory brain insufficiency and chronic cholecystopancreatitis. Analysis of concomitant illnesses revealed no between-group differences (Supplementary Table 1).

Concomitant medications assessed included angiotensin converting enzyme inhibitor, diuretics, anti-aggregants, beta-blockers, blockers of AT1 receptors, calcium channel blockers, polyferment drugs, sugar-lowering drugs, homeopathic remedies, metabolic drugs, antagonists of alpha 1 adrenergic receptors, antidepressants, anti-migraine medications, ophthalmic drugs, gastrointestinal medications, nonsteroidal anti-inflammatory drugs, nitrates, nootropic drugs, acid-dependent diseases medications, drugs used in the treatment of cough and colds, sleep and sedative medications, phytotherapeutics, anti-prostate hyperplasia drugs, drugs used in the treatment of musculoskeletal system diseases and drugs for vascular therapy. Analysis of concomitant illnesses revealed no between-group differences (Supplementary Table 2).

### Primary outcome

**Fig. 2:**
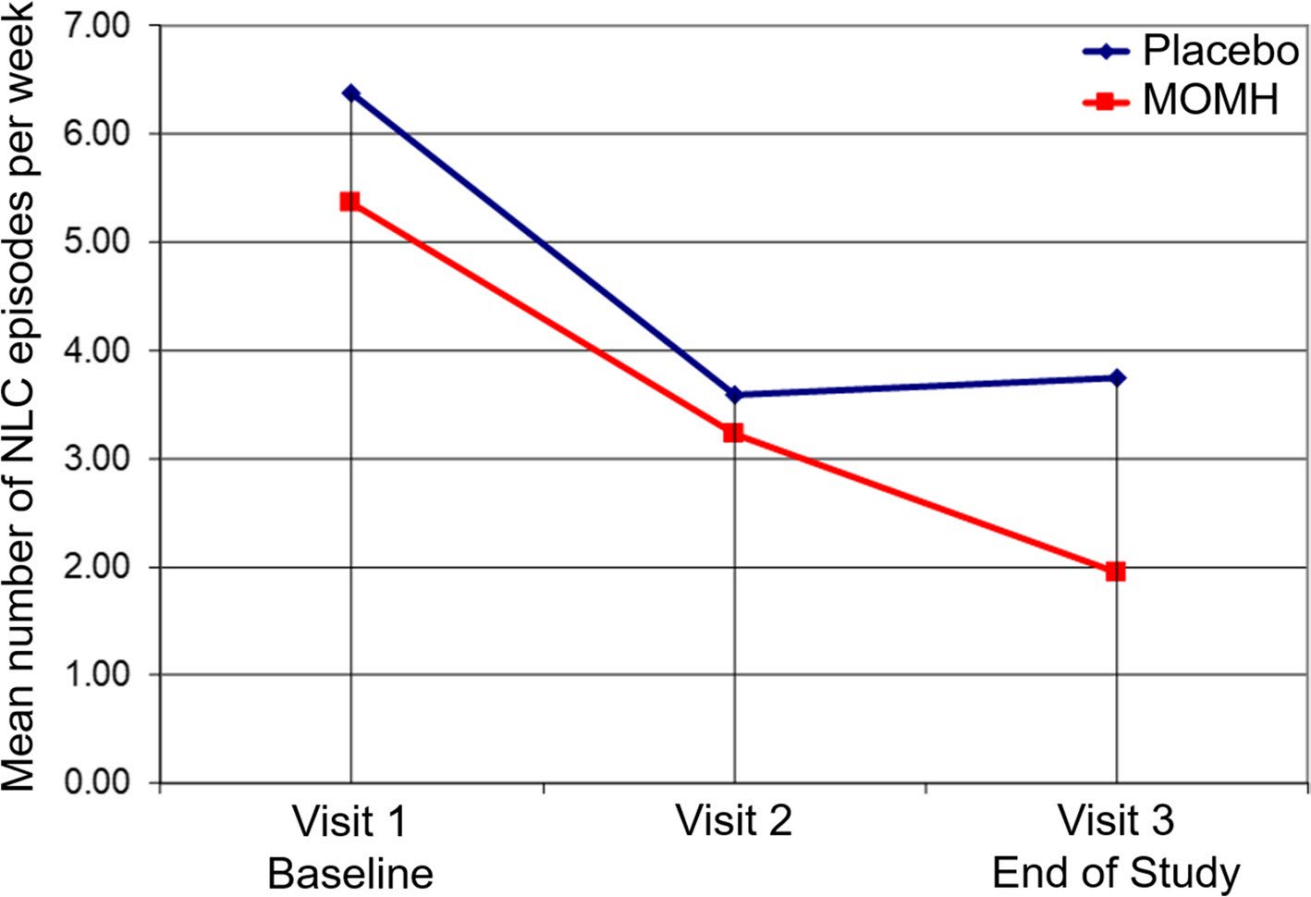
The Change in the Number of NLC Episodes During the Study. Mean number of NLC episodes per week as calculated at each study visit is presented for each group (placebo (n = 87) – blue; MOMH (n = 86) – red)

The primary efficacy analysis was performed on the number of NLC episodes per week calculated at each study visit. The dynamics of the mean number of NLC episodes per week over the study period are presented in Fig. 2.

A significant change in the number of NLC episodes per week was observed for both groups at Visit 2 as compared to Baseline (means for the placebo group: 6.4 vs 3.6, *p* < 0.001; means for MOMH group: 5.4 vs 3.2, p < 0.001) and at Visit 3 as compared to Baseline (means for the placebo group: 6.4 vs 3.7, p < 0.001; means for MOMH group: 5.4 vs 1.9, p < 0.001). The magnitude of the reduction in NLC frequency was compared between the groups using ANOVA on ranks and contrasts. There was no significant between-group difference in the magnitude of NLC frequency reduction 30 days after treatment initiation (Visit 2, *p* = 0.099). However, when assessed 60 days after treatment initiation, a significant between-group difference in the magnitude of NLC frequency reduction was revealed (Visit 3, *p* = 0.005), indicating a larger effect in the MOMH group.

### Secondary outcomes

**Fig. 3:**
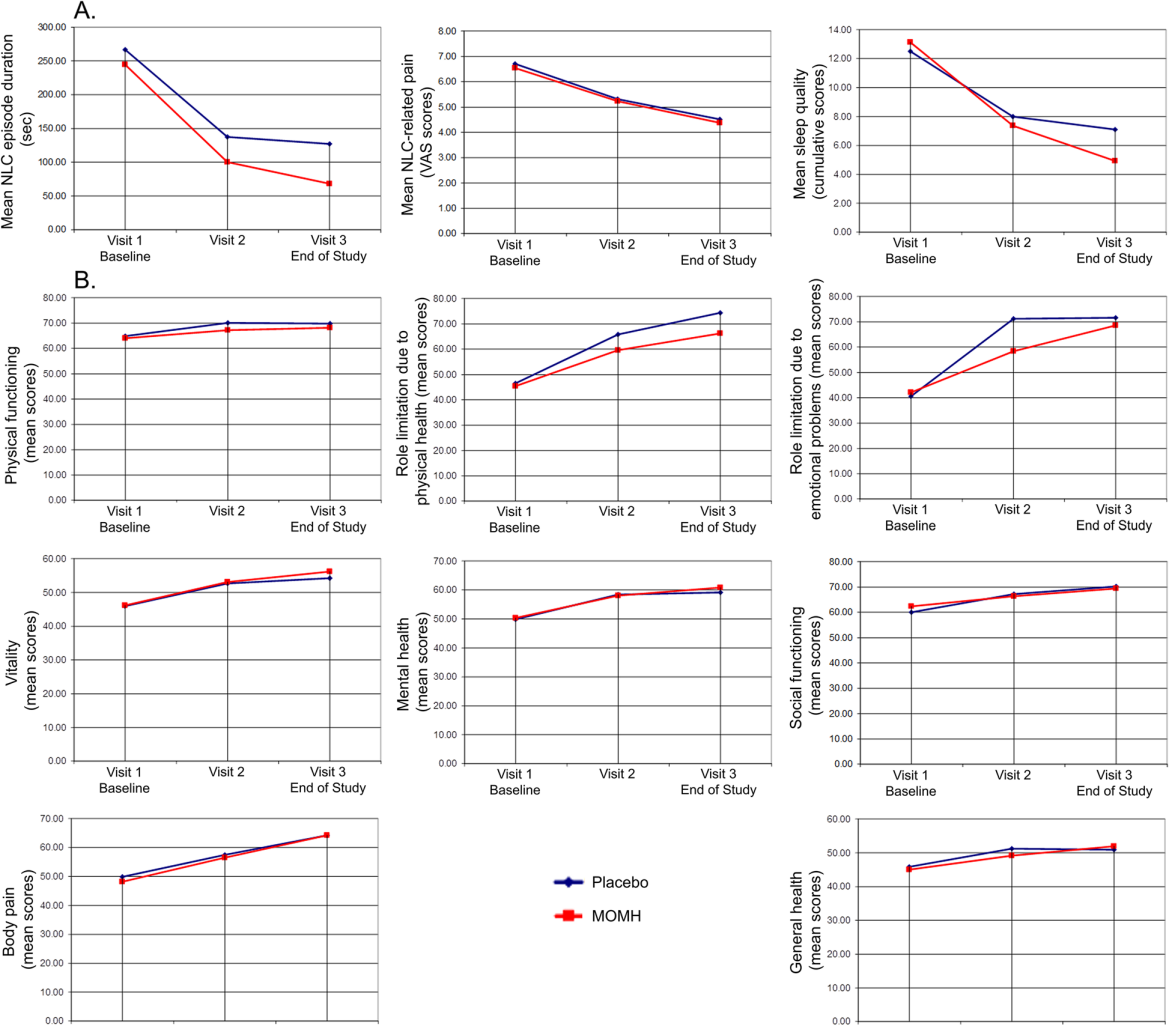
Analysis of the Secondary Efficacy Parameters. Secondary efficacy parameters are presented as calculated at each study visit for each study group (Placebo (N = 87) – blue, MOMH (N = 86) – red). A. Mean NLC episode duration (left, MOMH N = 86), NLC-related pain (middle), Sleep quality (right). Reduction in these parameters indicates improvement. B. Quality of life parameters – Physical functioning (top left), Role limitation due to physical health (top middle), Role limitation due to emotional problems (top right), Vitality (middle left), Mental health (middle), Social functioning (middle right), Body pain (bottom left), General health (bottom right). Increased values indicate improvement

The duration of the NLC episodes, the severity of NLC-induced pain, the quality of sleep and the quality of life were studied as part of the secondary efficacy analysis. The dynamics of these parameters as measured at each of the study visits are presented in Fig. 3.

A significant reduction in the NLC episode duration was found for both groups at Visit 2 as compared to Baseline (means for the placebo group: 137.4 vs 266.5, *p* < 0.001; means for MOMH group: 99.9 vs 244.5, p < 0.001) and at Visit 3 as compared to Baseline (means for the placebo group:127.2 vs 266.5 *p* < 0.001; means for MOMH group: 67.9 vs 244.5, p < 0.001). A marginally significant between-group difference in the magnitude of reduction in the NLC episode duration from Baseline to Visit 2 (*p* = 0.057) and a significant between-group difference from Baseline to Visit 3 (*p* = 0.004) were identified, indicating a larger reduction in the mean NLC episode duration in MOMH group.

A significant reduction in pain was observed for both groups at Visit 2 as compared to Baseline (means for the placebo group: 5.3 vs 6.7, *p* < 0.001; means for MOMH group: 5.3 vs 6.6, *p* < 0.001) and at Visit 3 as compared to Baseline (means for the placebo group: 4.5 vs 6.7, *p* < 0.001; means for MOMH group: 4.4 vs 6.6, p < 0.001). Between-group comparisons of the magnitude of change in the pain scores revealed no significant between-group differences (*p* = 0.954), indicating that treatment with MOMH and treatment with placebo had similar impact on pain.

Sleep quality was assessed, and a significant improvement was identified for both groups at Visit 2 as compared to Baseline (means for the placebo group: 8.0 vs 12.5, *p* < 0.001; means for MOMH group: 7.2 vs 13.1, p < 0.001) and at Visit 3 as compared to Baseline (means for the placebo group: 7.1 vs 12.5, p < 0.001; means for MOMH group: 5.0 vs 13.0, p < 0.001). The magnitude of improvement from Baseline to Visit 2 was significantly different between the groups (*p* = 0.049), as was the magnitude of improvement from Baseline to Visit 3 (p < 0.001), indicating larger improvement in MOMH group.

The quality-of-life parameters significantly improved for both groups at Visit 2 and Visit 3 as compared to Baseline (*p* < 0.045 for all). The magnitude of improvement in all parameters was compared between the groups. Role limitation due to physical health (RP) and Role limitation due to emotional problems (RE) were the only parameters for which a significant between-group difference was observed (RP (from Baseline to Visit 3): *p* = 0.017; RE (from Baseline to Visit 2): *p* = 0.021). This difference indicated larger improvement in MOMH group.

One subject from the placebo group and 6 subjects from the MOMH group withdrew their consent during the study period, indicating a relatively low dropout rate and high tolerability.

### Safety/tolerability and adverse effects

177 subjects (placebo group *N* = 88; MOMH group *N* = 89) received at least 1 dose of the study treatment and were included in the analysis of safety and tolerability. There were no deaths or serious adverse events during the study. 4 subjects from the placebo group reported of having adverse events that included fatigue, headache, nausea, diarrhea, and muscle twitching. No adverse events were reported in the MOMH group.

### Post-hoc analyses

Following the database lock, a by-site post-hock analysis of the efficacy variables was carried out to assess site-specific outcomes. It was discovered that the results obtained from Site 1 demonstrated opposite dynamics (placebo superior to MOMH) as compared to the other six sites. To learn about the potential sources for this difference, the collected data were divided into two subsets. Subset 1 included the data obtained from Site 1; Subset 2 included the data obtained from all the other sites. Baseline characteristics were compared between the two subsets of results and are presented in Table 2. Significant between-group differences were found in gender distribution, which indicated that Subset 1 was more equally gender-distributed whereas female participants constituted the majority of Subset 2 subjects. In addition, Subset 1 participants were younger, taller and had significantly higher SBP and DBP (Table 2).

**Table 2:** Baseline Characteristics by Subset

	Subset 1 (*N* = 44)	Subset 2 (*N* = 129)	P Value
Demographics			
Gender Female n (%)	26 (59.1)	103 (79.8)	**0.009**
Age (years) mean (±SD)	50.0 (±6.1)	59.7 (±10.5)	**< 0.001**
Weight (kg) mean (±SD)	75.6 (±16.1)	74.7 (±12.4)	0.638
Height (cm) mean (±SD)	171.4 (±12.5)	166.1 (±7.7)	**0.001**
BMI (kg/m^2^) mean (±SD)	25.5 (±3.4)	27.1 (±4.4)	0.108
Physiological parameters			
Systolic blood pressure (mmHg) mean (±SD)	134.2 (±11.4)	127.7 (±10.2)	**< 0.001**
Diastolic blood pressure (mmHg) mean (±SD)	83.4 (±5.2)	78.2 (±6.5)	**< 0.001**
Heart rate (beats/min) mean (±SD)	70.6 (±5.0)	71.8 (±6.7)	0.429
Baseline efficacy parameters			
NLC frequency (num/week) mean (±SD)	3.9 (±0.8)	6.6 (±7.9)	**0.003**
NLC duration (sec/week) mean (±SD)	48.7 (±12.9)	326.1 (±244.5)	**< 0.001**
NLC pain (mean VAS/week) mean (±SD)	6.7 (±0.9)	6.6 (±1.6)	0.686
Sleep quality (mean cumulative score/week) mean (±SD)	13.0 (±2.9)	12.7 (±3.7)	0.627

*BMI* body mass index, *NLC* nocturnal leg cramps, *SD* standard deviation, *VAS* visual analogue scale

In terms of the baseline efficacy parameters, Subset 1 subjects experienced significantly fewer NLC episodes which, overall, persisted substantially less time as compared to Subset 2 subjects.

Between-subset comparison of concomitant medications revealed that higher proportion of Subset 1 subjects were treated with anti-aggregants (36.4% in Subset 1 vs 14.0% in Subset 2, *p* = 0.002), polyferment drugs (11.4% in Subset 1 vs 0.8% in Subset 2, *p* = 0.004) and medications for gastrointestinal disorders (6.8% in Subset 1 vs 0% in Subset 2, *p* = 0.016), whereas the proportion of subjects treated with angiotensin converting enzyme inhibitor was higher in Subset 2 (45.7% in Subset 2 vs 27.3% in Subset 1, p = 0.002). Other concomitant medications assessed included betablockers, diuretics, angiotensin II receptor blockers, medications used in cough and colds, homeopathic products, anti-prostate hyperplasia medications, acid-dependent disease remedies, antagonists of alpha 1 adrenergic receptors, antidepressants, anti-migraine remedies, medications used in the treatment of musculoskeletal system diseases, medications used in gynecology, ophthalmic medications, metabolic medications, nonsteroidal anti-inflammatory medications, nitrates, nootropics, medications for vascular therapy, sedative-hypnotic drugs, phyto-therapeutics and sugar lowering medications. No significant differences were observed.

Comparison of comorbidities showed higher proportion of heart failure (27.3% in Subset 1 vs 0% in Subset 2, *p* < 0.0001), chronic prostatitis (15.9% in Subset 1 vs 0.8% in Subset 2, p < 0.0001), chronic pancreatitis (11.4% in Subset 1 vs 1.6% in Subset 2, *p* = 0.012), chronic gastritis (11.4% in Subset 1 vs 0.8% in Subset 2, *p* = 0.004), chronic tonsillitis (13.6% in Subset 1 vs 0.8% in Subset 2, *p* = 0.001), chronic cystitis (9.1% in Subset 1 vs 0% in Subset 2, p = 0.004) and myopia (6.8% in Subset 1 vs 0% in Subset 2, *p* = 0.015) in Subset 1 subjects. Cardiosclerosis (10.9% in Subset 2 vs 0% in Subset 1, *p* = 0.022) and chronic cholecystitis (9.3% in Subset 2 vs 0% in Subset 1, *p* < 0.039) were diagnosed in higher proportion of Subset 2 subjects as compared to Subset 1. Other comorbidities assessed included hypertension, ischemic heart disease, osteochondrosis, arthrosis, chronic bronchitis, diabetes mellitus, angina pectoris, stomach ulcer, chronic pyelonephritis, hypertensive heart, neurocirculatory dystonia of the cardiac type, mastopathy, chronic cholecystopancreatitis, adenoma of prostate, arthritis, cerebral atherosclerosis, bronchial asthma, varicose disease of the lower extremities, tension headache, eczema, dizziness, insomnia, history of ischemic stroke, kidney cyst, climacteric vegetative disorders, migraine, breast myoma, pre-diabetes (impaired glucose tolerance), cataract, urinogenic diathesis, anxiety disorder, Parkinson’s disease, chronic dyscirculatory brain insufficiency, chronic gastroduodenitis, cerebral atherosclerosis, chronic tonsillopharyngitis, chronic sinusitis and chronic glomerulonephritis. No significant differences were observed.

These results indicate that Subset 1 subjects had a somewhat more severe profile in terms of concomitant medications and comorbidities as compared to Subset 2 subjects.

To assess the impact of Site 1 data on the outcomes we reanalyzed the results with Subset 1 data entirely excluded.

MOMH and placebo groups of Subset 2 were compared for characteristics and baseline efficacy outcomes. The results are presented in Table 3.

**Table 3:** Baseline Characteristics of Subset 2 data by Group

	MOMH (*N* = 64)	Placebo (*N* = 65)	P Value
Demographics			
Gender Female n (%)	52 (81.3)	51 (78.5)	0.827
Age (years) mean (±SD)	60.4 (±10.9)	59.1 (±10.1)	0.517^a^
Weight (kg) mean (±SD)	75.1 (±11.8)	74.3 (±13.1)	0.583
Height (cm) mean (±SD)	165.9 (±7.7)	166.2 (±7.8)	0.278
BMI (kg/m^2^) mean (±SD)	27.3 (±4.1)	26.9 (±4.8)	0.395^a^
Physiological parameters			
Systolic blood pressure (mmHg) mean (±SD)	127.6 (±10.9)	127.7 (±9.6)	0.826^a^
Diastolic blood pressure (mmHg) mean (±SD)	78.8 (±6.7)	77.5 (±6.2)	0.154^a^
Heart rate (beats/min) mean (±SD)	72.0 (±6.9)	71.7 (±6.6)	0.749^a^
Baseline efficacy parameters			
NLC frequency (num/week) mean (±SD)	5.8 (±5.7)	7.3 (±9.5)	0.753^a^
NLC duration (sec/week) mean (±SD)	311.4 (±242.9)	340.7 (±247.1)	0.410^a^
NLC pain (mean VAS/week) mean (±SD)	6.5 (±1.6)	6.8 (±1.5)	0.252
Sleep quality (mean cumulative score/week) mean (±SD)	13.0 (±3.7)	12.5 (±3.8)	0.451
Quality of life subscales:			
- Physical functioning	58.6 (±24.2)	60.2 (±26.9)	0.934^a^
- Role limitation due to physical health	38.3 (±44.5)	40.8 (±43.4)	0.616^a^
- Role limitation due to emotional problems	37.0 (±44.9)	33.9 (±43.1)	0.696^a^
- Vitality	45.4 (±14.2)	45.5 (±12.4)	0.783^a^
- Mental health	49.2 (±13.8)	49.2 (±12.0)	0.879^a^
- Social functioning	60.4 (±15.7)	57.7 (±15.3)	0.457^a^
- Body Pain	46.0 (±18.2)	46.0 (±17.7)	0.921^a^
- General health	44.3 (±13.1)	45.4 (±8.8)	0.580^a^

*BMI* body mass index, *MOMH* magnesium oxide monohydrate, *NLC* nocturnal leg cramps, *SD* standard deviation, *VAS* visual analogue scale

Note: Variables marked with ^a^ were not normally distributed. ANOVA and ANCOVA on ranks were performed for comparisons of these variables

There was no difference in the demographics and physiological parameters between the groups. Importantly, no between-group differences in any of the baseline efficacy parameters were identified.

Analysis of comorbidities revealed no difference between the groups, except for hypertension which had higher incidence in MOMH group (73.4% vs 53.9%, *p* = 0.028). There was no between-group difference in concomitant medications.

All efficacy parameters significantly improved in both groups from Baseline to Visit 2 (*p* < 0.02 for all) and from Baseline to Visit 3 (*p* < 0.01 for all). Between-group comparisons of the magnitude of improvement revealed a significant difference in favor of the MOMH treatment in the primary efficacy parameter of NLC episodes number per week (Baseline to Visit 2: *p* = 0.007; Baseline to Visit 3: *p* < 0.001) and in the following secondary efficacy parameters: NLC duration (Baseline to Visit 2: *p* = 0.003; Baseline to Visit 3: p < 0.001), NLC pain (Baseline to Visit 2: *p* = 0.023; Baseline to Visit 3: p < 0.001) and sleep quality (Baseline to Visit 2: p < 0.001; Baseline to Visit 3: p < 0.001). These results indicate that when Site 1 data were excluded, a more robust advantage for MOMH treatment over placebo was observed.

## Discussion

Magnesium plays an important role in hundreds of metabolic reactions, including those that govern muscle function [24–26]. The threshold of axon stimulation is decreased, and nerve conduction velocity is increased when serum magnesium is reduced, leading to an increase in the excitability of muscles and nerves. The cellular basis for these changes is increased intracellular calcium content. Magnesium deficiency leads to neuronal excitability and enhances neuromuscular transmission [3, 13, 27, 28] and its substitution has been shown to be effective in eclampsia-related seizures [25, 28–30]. Due to these characteristics some authors suggested a beneficial role for magnesium in NLC. From the functional perspective, experimental studies suggest that magnesium administration might enhance glucose uptake and limit lactate accumulation in the skeletal muscle leading to reduced pain following muscle contraction [31]. In line with this suggestion, magnesium has been shown to improve vascular endothelial function [32, 33], cardiac fitness and walking time [34, 35], perhaps via vasodilatory effect. In addition, magnesium has been shown to potentially reduce damage caused by oxygen free radicals and decrease platelet hyperactivity, such as adhesion and aggregation [36, 37]. However, the specific role of magnesium supplementation in preventing and/or treating muscle cramps remains unclear. No current treatments for NLC have been proven both safe and effective [1].

Though magnesium is widely prescribed in Europe and across the world for NLC treatment [3], uncertainty remains as to whether it is beneficial [3, 5, 38]. We carried out a multi-center, randomized, placebo-controlled trial with the aim of assessing the efficacy and safety of magnesium dietary supplement MOMH, in treating NLC. We found that following 60 days of daily treatment, subjects who received MOMH showed a significantly larger improvement in the number of NLC episodes per week, NLC duration per week and sleep quality, as compared to placebo, indicating superiority of MOMH. A favorable safety profile was demonstrated as well, as no adverse events were reported in the MOMH group.

Our results are in line with the results obtained in a double-blind, randomized, placebo-controlled study of pregnant women with NLC [11]. Two previously-published cross-over studies [13, 14] demonstrated a lack of beneficial effect for magnesium citrate supplements in treating NLC in older adults. We interpret the lack of beneficial effect as stemming from a potentially low intracellular absorption of magnesium citrate. A recent study investigated the effect of supplemental oral MOMH vs magnesium citrate, on intracellular magnesium levels in healthy subjects [18]. The authors showed that oral MOMH administration led to significantly higher intracellular magnesium levels as compared with magnesium citrate. Another potential explanation for the lack of beneficial impact could be a significant period-bias effect, which implies overall time-induced improvement unrelated to a specific treatment.

Oral administration of MOMH to individuals with NLC was tested in a separate study and no significant effects were found [19]. We believe that this null result stems from a high dropout rate (~ 47%) and a relatively short duration of the treatment period (4 weeks) implemented in this study [19].

Taking into account the favorable cellular accumulation potential of MOMH and the evidence suggesting that longer administration periods are required [18], the current study was conducted with MOMH supplement administered for 60 days and superiority over placebo was demonstrated.

### Study limitations

Although the placebo and the MOMH group were similar in terms of their baseline characteristics, including demographic and health-related factors, 3 subgroups could be identified with respect to comorbidities: 1. Cardiovascular patients (CVD). 2. Gastrointestinal patients (GI). 3. Healthy subjects. CVD and GI patients could have impaired build-up of magnesium levels due to absorption and/or excretion problems, thus masking the positive impact of MOMH on NLC. These patients could have benefited from higher MOMH doses. Further research is required to assess the possibility of administering higher doses of MOMH.

Another limitation is the lack of concurrent tracking of intracellular magnesium levels to assess the direct relationship between intracellular magnesium and the NLC treatment efficacy.

The duration of the treatment period could still have not been sufficiently long to produce a robust effect, generalized across all the tested parameters. Future studies should consider longer treatment periods.

Additional factors that could have potentially impacted the results, but were not monitored, include dietary intake and physical activity. These factors should be taken into account in future studies.

NLC diagnosis based on self-report as well as subjective evaluations of the secondary efficacy parameters (pain, sleep quality and quality of life), although accepted as valid tools and are widely used in the clinical research, still pose a limitation to the study.

## Conclusions

Our results demonstrate the superiority of MOMH over placebo in treating NLC with respect to the number of NLC episodes, their duration and sleep quality. The current study demonstrates that MOMH can provide a clinical solution to non-pregnant individuals who suffer from NLC.

## Supplementary Information

**Additional file 1: Supplementary Table 1**: Concomitant Diseases.

**Additional file 2: Supplementary Table 2**: Concomitant Medications.

## Data Availability

The datasets used and analyzed during the current study are available from the corresponding author on reasonable request.

## Abbreviations

ANCOVA: Analysis of Covariance
ANOVA: Analysis of Variance
BMI: Body Mass Index
CVD: Cardiovascular patients
DBP: Diastolic Blood Pressure
GI: Gastrointestinal patients
MOMH: Magnesium Oxide Monohydrate
NLC: Nocturnal Leg Cramps
SBP: Systolic Blood Pressure
SF-36: 36-Item Short Form Health Survey
VAS: Visual Analogue Scale
