# Supplementary material for: A randomized, double-blind, placebo-controlled, multicenter study assessing the efficacy of magnesium oxide monohydrate in the treatment of nocturnal leg cramps
Source: Nutr J. 2021 Oct 31;20:90. doi: 10.1186/s12937-021-00747-9 (PMC8559389; doi:10.1186/s12937-021-00747-9)
Supplement: Supplementary file 1 — Additional file 1: Supplementary Table 1: Concomitant Diseases. [file 12937_2021_747_MOESM1_ESM.docx]

**Supplementary Table 1: Concomitant Diseases**

| Disease | MOMH group | | Placebo group | | P-value* |
| --- | --- | --- | --- | --- | --- |
|  | n** | % | n*** | % |  |
| Hypertension | 53 | 61.63 | 46 | 52.87 | 0.283 |
| Coronary artery disease | 13 | 15.12 | 7 | 8.05 | 0.161 |
| Cardiosclerosis | 8 | 9.30 | 6 | 6.90 | 0.590 |
| Osteochondrosis | 8 | 9.30 | 8 | 9.20 | 1.000 |
| Heart failure | 5 | 5.81 | 7 | 8.05 | 0.766 |
| Angina pectoris | 5 | 5.81 | 1 | 1.15 | 0.117 |
| Chronic tonsillitis | 4 | 4.65 | 3 | 3.45 | 0.720 |
| Chronic cholecystitis | 4 | 4.65 | 8 | 9.20 | 0.370 |
| Arthrosis | 3 | 3.49 | 6 | 6.90 | 0.496 |
| Chronic pancreatitis | 3 | 3.49 | 4 | 4.60 | 1.000 |
| Chronic prostatitis | 3 | 3.49 | 5 | 5.75 | 0.720 |
| Diabetes mellitus | 3 | 3.49 | 3 | 3.45 | 1.000 |
| Stomach ulcer | 2 | 2.33 | 1 | 1.15 | 0.621 |
| Hypertensive heart | 2 | 2.33 | 0 | 0.00 | 0.246 |
| Chronic sinusitis in remission stage | 2 | 2.33 | 2 | 2.30 | 1.000 |
| Chronic gastritis | 2 | 2.33 | 4 | 4.60 | 0.682 |
| Chronic pyelonephritis | 2 | 2.33 | 1 | 1.15 | 0.621 |
| Chronic cystitis in remission stage | 2 | 2.33 | 1 | 1.15 | 0.621 |
| Adenoma of prostate | 1 | 1.16 | 0 | 0.00 | 0.497 |
| Bronchial asthma | 1 | 1.16 | 0 | 0.00 | 0.497 |
| Varicose disease of the lower extremities | 1 | 1.16 | 0 | 0.00 | 0.497 |
| Tension headache | 1 | 1.16 | 0 | 0.00 | 0.497 |
| Dizziness | 1 | 1.16 | 0 | 0.00 | 0.497 |
| Insomnia | 1 | 1.16 | 0 | 0.00 | 0.497 |
| Migraine | 1 | 1.16 | 0 | 0.00 | 0.497 |
| Myopia | 1 | 1.16 | 1 | 1.15 | 1.000 |
| Neurocirculatory dystonia of the cardiac type | 1 | 1.16 | 1 | 1.15 | 1.000 |
| Cataract | 1 | 1.16 | 0 | 0.00 | 0.497 |
| Anxiety disorder | 1 | 1.16 | 0 | 0.00 | 0.497 |
| Fibroscopic mastopathy | 1 | 1.16 | 1 | 1.15 | 1.000 |
| Parkinson’s disease | 1 | 1.16 | 0 | 0.00 | 0.497 |
| Chronic bronchitis | 1 | 1.16 | 5 | 5.75 | 0.211 |
| Chronic gastroduodenitis | 1 | 1.16 | 3 | 3.45 | 0.621 |
| Chronic glomerulonephritis, remission stage | 1 | 1.16 | 0 | 0.00 | 0.497 |
| Chronic tonsillopharingitis, remission stage | 1 | 1.16 | 0 | 0.00 | 0.497 |
| Cerebral atherosclerosis | 0 | 0.00 | 2 | 2.30 | 0.497 |
| Post-onset (ischemic stroke in 2005)  Encephalopathy | 0 | 0.00 | 1 | 1.15 | 1.000 |
| Kidney cyst | 0 | 0.00 | 1 | 1.15 | 1.000 |
| Сlimacteric vegetative disorders | 0 | 0.00 | 1 | 1.15 | 1.000 |
| Constitutional-exogenous obesity, I degree. | 0 | 0.00 | 1 | 1.15 | 1.000 |
| Breast myoma | 0 | 0.00 | 1 | 1.15 | 1.000 |
| Pre-diabetes (Impairment glucose tolerance) | 0 | 0.00 | 1 | 1.15 | 1.000 |
| Reactive arthritis | 0 | 0.00 | 1 | 1.15 | 1.000 |
| Urinogenic diathesis | 0 | 0.00 | 1 | 1.15 | 1.000 |
| Eczema dyshidrotic, chronic | 0 | 0.00 | 1 | 1.15 | 1.000 |
| Chronic dyscirculatory brain insufficiency | 0 | 0.00 | 1 | 1.15 | 1.000 |
| Chronic cholecystopancreatitis | 0 | 0.00 | 3 | 3.45 | 0.246 |
| * Calculated by Fisher's Exact Test.  ** 86 = 100 % (number of patients in group, multiple entries were possible)  *** 87 = 100 % (number of patients in group, multiple entries were possible) | | | | | |
