# Supplementary material for: A randomized, double-blind, placebo-controlled, multicenter study assessing the efficacy of magnesium oxide monohydrate in the treatment of nocturnal leg cramps
Source: Nutr J. 2021 Oct 31;20:90. doi: 10.1186/s12937-021-00747-9 (PMC8559389; doi:10.1186/s12937-021-00747-9)
Supplement: Supplementary file 2 — Additional file 2: Supplementary Table 2: Concomitant Medications. [file 12937_2021_747_MOESM2_ESM.docx]

**Supplementary Table 2: Concomitant Medications**

| Pharmacological group | MOMH group | | Placebo group | | p-value* |
| --- | --- | --- | --- | --- | --- |
|  | **n**** | **%** | **n***** | **%** |  |
| Angiotensin converting enzyme inhibitor | 35 | 40.70 | 36 | 41.38 | 1.000 |
| Diuretics | 20 | 23.26 | 16 | 18.39 | 0.459 |
| Antiagregants | 19 | 22.09 | 15 | 17.24 | 0.450 |
| Beta-blockers | 11 | 12.79 | 12 | 13.79 | 1.000 |
| Blockers of AT1 receptors | 11 | 12.79 | 4 | 4.60 | 0.063 |
| Calcium channel blockers | 8 | 9.30 | 5 | 5.75 | 0.404 |
| Polyferment drugs | 3 | 3.49 | 3 | 3.45 | 1.000 |
| Sugar-lowering drugs | 3 | 3.49 | 4 | 4.60 | 1.000 |
| Homeopathic remedies | 2 | 2.33 | 1 | 1.15 | 0.621 |
| Metabolic drugs | 2 | 2.33 | 0 | 0.00 | 0.246 |
| Antagonists of alpha 1 adrenergic receptors | 1 | 1.16 | 0 | 0.00 | 0.497 |
| Antidepressants | 1 | 1.16 | 0 | 0.00 | 0.497 |
| Anti-migraine remedies | 1 | 1.16 | 0 | 0.00 | 0.497 |
| Ophthalmic drugs | 1 | 1.16 | 0 | 0.00 | 0.497 |
| Gastrointestinal remedies | 1 | 1.16 | 2 | 2.30 | 1.000 |
| Nonsteroidal anti-inflammatory drugs | 1 | 1.16 | 0 | 0.00 | 0.497 |
| Nitrates | 1 | 1.16 | 1 | 1.15 | 1.000 |
| Nootropic drugs | 1 | 1.16 | 1 | 1.15 | 1.000 |
| Acid-dependent diseases remedies | 1 | 1.16 | 0 | 0.00 | 0.497 |
| Drugs used at cough and colds | 1 | 1.16 | 1 | 1.15 | 1.000 |
| Sleep and sedative | 1 | 1.16 | 0 | 0.00 | 0.497 |
| Phytotherapeutics | 1 | 1.16 | 0 | 0.00 | 0.497 |
| Anti-prostate hyperplasia drugs | 0 | 0.00 | 1 | 1.15 | 1.000 |
| Drugs used in musculoskeletal system diseases | 0 | 0.00 | 1 | 1.15 | 1.000 |
| Drugs used in gynecology | 0 | 0.00 | 1 | 1.15 | 1.000 |
| Drugs for vascular therapy | 0 | 0.00 | 1 | 1.15 | 1.000 |
| * Calculated by Fisher's Exact Test.  ** 86 = 100 % (number of patients in group, multiple entries were possible)  *** 87 = 100 % (number of patients in group, multiple entries were possible) | | | | | |
